# Use-Wear Patterns on Wild Macaque Stone Tools Reveal Their Behavioural History

**DOI:** 10.1371/journal.pone.0072872

**Published:** 2013-08-16

**Authors:** Michael Haslam, Michael D. Gumert, Dora Biro, Susana Carvalho, Suchinda Malaivijitnond

**Affiliations:** 1 Research Laboratory for Archaeology and the History of Art, University of Oxford, Oxford, United Kingdom; 2 Division of Psychology, School of Humanities and Social Sciences, Nanyang Technological University, Singapore, Singapore; 3 Department of Zoology, University of Oxford, Oxford, United Kingdom; 4 Primate Research Unit, Department of Biology, Chulalongkorn University, Bangkok, Thailand; Université de Strasbourg, France

## Abstract

Burmese long-tailed macaques (*Macaca fascicularis aurea*) are one of a limited number of wild animal species to use stone tools, with their tool use focused on pounding shelled marine invertebrates foraged from intertidal habitats. These monkeys exhibit two main styles of tool use: axe hammering of oysters, and pound hammering of unattached encased foods. In this study, we examined macroscopic use-wear patterns on a sample of 60 wild macaque stone tools from Piak Nam Yai Island, Thailand, that had been collected following behavioural observation, in order to (i) quantify the wear patterns in terms of the types and distribution of use-damage on the stones, and (ii) develop a Use-Action Index (UAI) to differentiate axe hammers from pound hammers by wear patterns alone. We used the intensity of crushing damage on differing surface zones of the stones, as well as stone weight, to produce a UAI that had 92% concordance when compared to how the stones had been used by macaques, as observed independently prior to collection. Our study is the first to demonstrate that quantitative archaeological use-wear techniques can accurately reconstruct the behavioural histories of non-human primate stone tools.

## Introduction

The developing field of primate archaeology [1]–[8] aims to identify and analyse artefacts used and accumulated by primates from outside the human lineage. Primate archaeology benefits palaeoanthropological research by providing comparative data for the evolution of hominin technology and landscape use, in addition to reconstructing the behavioural history of wild primates through the recovery and recording of tools used by these animals [9]–[12]. Stone tools are particularly important as their durability increases the likelihood that they will survive in the archaeological record. The only primates currently known to use stone tools in the wild are western chimpanzees (*Pan troglodytes verus*), bearded and yellow-breasted capuchin monkeys (*Sapajus libidinosus* and *S. xanthosternos*), and Burmese long-tailed macaques (*Macaca fascicularis aurea*) [13]–[16]. Although widely separated both geographically and phylogenetically in the ape, New World and Old World monkey lineages respectively, all these species use stones to pound open encased foods. Other wild species that use stone tools, including digger wasps (*Ammophila* and *Sphex* sp.), sea otters (*Enhydra lutis*) and Egyptian vultures (*Neophron percnopterus*) [17], also use these tools as pounding implements.

Use-wear analyses can play an important role in characterising stone tools used by non-human primates, as these tools are not deliberately modified by the animals and use-damage is a primary means of distinguishing natural from utilised stones [13]. Previous studies have reported pits on tool surfaces that result from attrition during repeated pounding impacts [3], [4], [6], [18], [19], although the process of pit formation has not been quantified and other types of use-wear are not systematically described [1], [20]. Pits found on Middle Pleistocene artefacts from Israel have been attributed to hominin nut-cracking activities [21], and damaged pounding tools have been described from the African Early Stone Age [22] and in experimental contexts [23]. To our knowledge there are no systematic published data on the types and surface distribution of use-wear on non-human primate tools, despite the potential value of such data as a means of (i) identifying tools and their use-history in the absence of direct observation, and (ii) interpreting the action, precision, and grips used by wild tool-using primates. Here, we report the first quantitative use-wear analysis of stone pounding tools used by wild Burmese long-tailed macaques (*Macaca fascicularis aurea*), the only known stone tool using wild Old World monkeys.

Burmese long-tailed macaques use stone tools as foraging aids to process marine invertebrates and plant parts in coastal areas and mangroves in Thailand and Myanmar [15], [20], [24], [25]. Their use of stones is only known to occur at a few sites, and where these macaques use stones as tools the behaviour is customary [26], following the definition of customary as ‘enacted regularly or predictably by all appropriate members of a group or population’ [27]. As a product of their regular tool use activity, these macaques leave large amounts of debris in the intertidal zone, including scarred tools and fragments of shelled marine organisms and seashore nuts. *M. f. aurea* stone tool use has been categorized into two basic tool-use styles, axe-hammering and pound-hammering, which were identified based on both physical characteristics of the stones and how the macaques used them [20]. Axe hammers are generally smaller and used with more rapid strikes, greater precision and more control than larger pound hammers. Axe-hammering also is largely directed at sessile oysters, while pound hammering is mainly used for processing non-sessile shelled items, such as gastropods, nuts and crabs [25]. Another difference is that damage found on axe hammers is more often associated with the pointed ends of the tool, while for pound hammers damage is found more frequently on the tool face [20]. Consequently, these two different usage patterns produce differing wear patterns on the stones.

Our aims in this study were to characterise the use-wear types and distribution on a sample of stone tools used by wild long-tailed macaques, and to develop analytical procedures that could reliably distinguish wear patterns characteristically produced on *M. f. aurea* stone tools used as axe or pound hammers. By matching use-wear patterns to behaviour, we aim to help researchers to identify and interpret macaque stone tools recovered during excavations or field surveys within areas currently or previously inhabited by long-tailed macaques.

## Methods

### Study site and subjects

**Figure 1:**
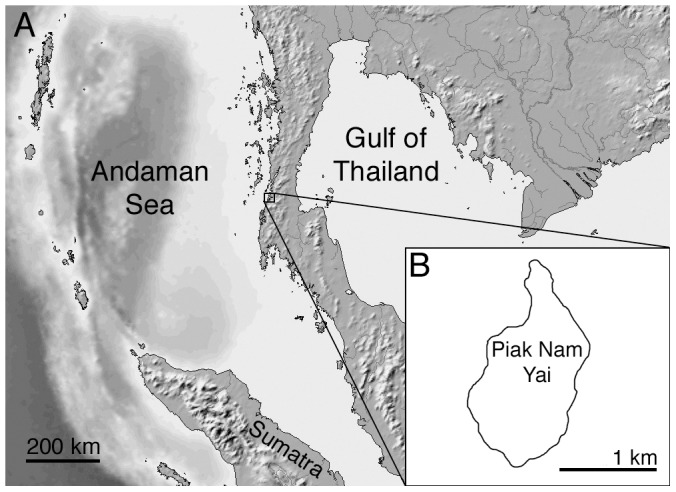
Location of the study site.

We studied stone tools used by wild Burmese long-tailed macaques on Piak Nam Yai (PNY) island, located in Laem Son National Park, Ranong Province, on the west coast of Thailand (N 9° 34–35’, E 98° 28’) [15], [20], [26], [28] (Figure 1). Tool-using macaques were first noted on PNY in early 2005, during a faunal survey assessing the impact of the major Indian Ocean tsunami of late 2004. PNY has an area of 1.7 km^2^ and consists of mountainous tropical forest surrounded by 5.4 km of coastline. The shoreline is subject to tidal fluctuations, and includes rocky shore, mangroves and a small sandy beach. Surveys in 2011 established that approximately 200 unhabituated wild macaques live on the island in nine groups [26]. These macaques forage daily in the intertidal regions of PNY for sessile and mobile marine invertebrates, and they often use stones to process these foods [25]. Macaque stone tool use is customary on PNY and 88% of the adult and adolescent monkeys on the island have been observed using stone tools [26]. They are unique among the known tool-using non-human primates in that they regularly process a wide variety of fauna using stone tools.

### Tool collection

**Figure 2:**
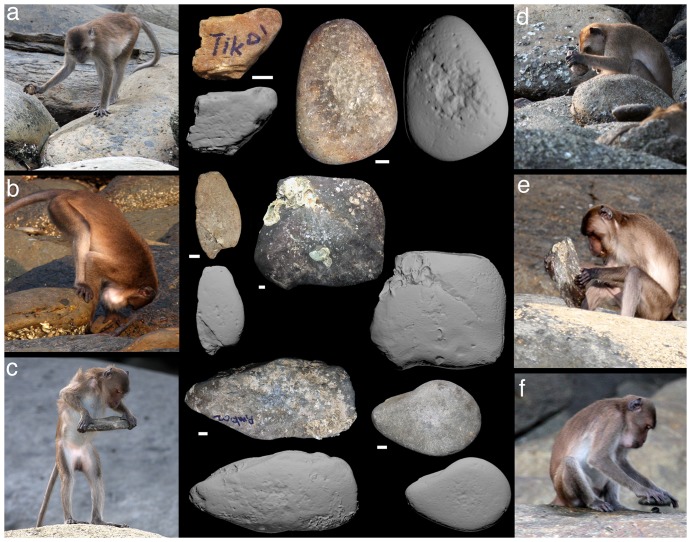
A selection of the analysed tools. Each tool is accompanied by a photo of the tool in use by a macaque prior to its collection. Each tool is represented by a photograph (with 1 cm scale) and a 3D scan of the same face, displaying use-wear. Tool codes are: (a) Tik01; (b) Che01; (c) Amb02; (d) Orc01; (e) Sln01; (f) Amb03. Macaque photos by MG, tool photos by MG and MH.

MG collected 60 stone tools from the rocky shores and mangroves of PNY between February and April 2011. The tool materials include sandstone, basalt, quartz, and siltstone. All tools were collected immediately following behavioural observations, conducted from a boat, of macaque tool use (Figure 2). MG identified the macaque that last used the tool in all cases, as well as the tool style employed and the food that was processed. Tools were selected for collection based on the opportunity of recovering the tool (i.e., the tool was left conspicuously after use and the boat was able to land). The macaques picked up the stones from among the naturally occurring stones on PNY, and the researchers did not influence their selection in any way. After collection, the stones were measured for physical characteristics, logged into a database, and put into storage for later analysis. All tools were photographed for archival purposes.

### Use-wear analysis

In May 2012 MH studied the collected tools for macroscopic use-wear. Microscopic use-wear was not examined. All use-wear data were collected blind, without MH having access to the observational data of MG. Previously, Gumert et al. [20] assessed stone tools used by Burmese long-tailed macaques as being damaged on their point, edge or face, or any combination of the three. The present study aimed to test whether indirect evidence from use-wear is able to accurately reconstruct the pounding techniques that were directly observed, and therefore we built on the previous work by including additional specificity and quantification of use-wear types and location on each tool. Two methods were used: (i) use-wear assessment that divided each artefact’s surface into ten zones and scored damage patterns within those zones, and (ii) 3D laser scanning that documented the types of use-damage preserved on the tools (see below for details of the recorded use-wear types). Laser scanning is becoming more common in archaeology for both data collection and quantitative analyses [29], [30], and the use of zones for interpreting the distribution of artefact functional evidence has previously proven valuable in both residue and use-wear analyses [31], [32].

**Figure 3:**
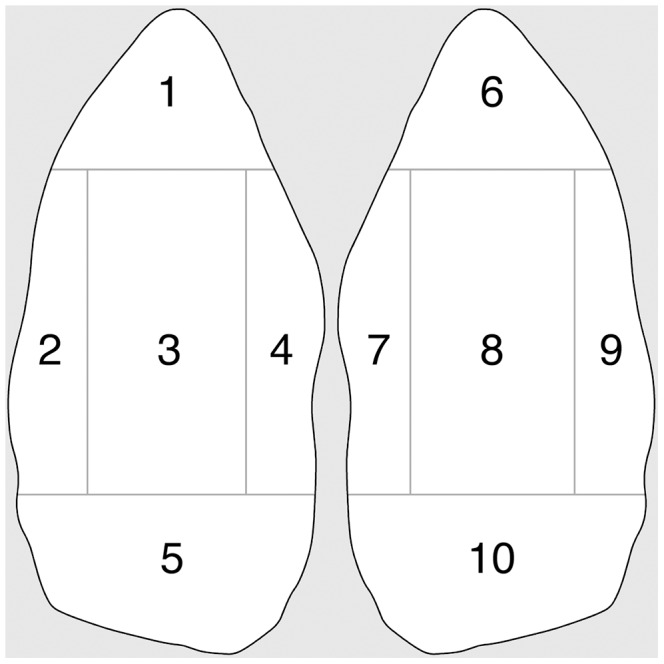
Schematic diagram of the tool zones.

Each artefact was oriented along its long axis with the narrowest end towards the top, with ten surface zones defined by dividing the tool at 25% and 75% of its length, and 25% and 75% of its width. These zones were numbered as shown in Figure 3. In turn these were clustered into four tool sections for ease of discussion. The narrower tool ‘point’ includes two zones (1, 6), the ‘edges’ comprise four zones (2, 4, 7, 9), the ‘base’ includes two zones (5, 10) at the opposite end of the tool from the point, and there are two ‘face’ zones (3, 8). Note that the previous study [20] treated both the ends of the tool long axis as ‘points’, and therefore did not include a ‘base’ category.

**Figure 4:**
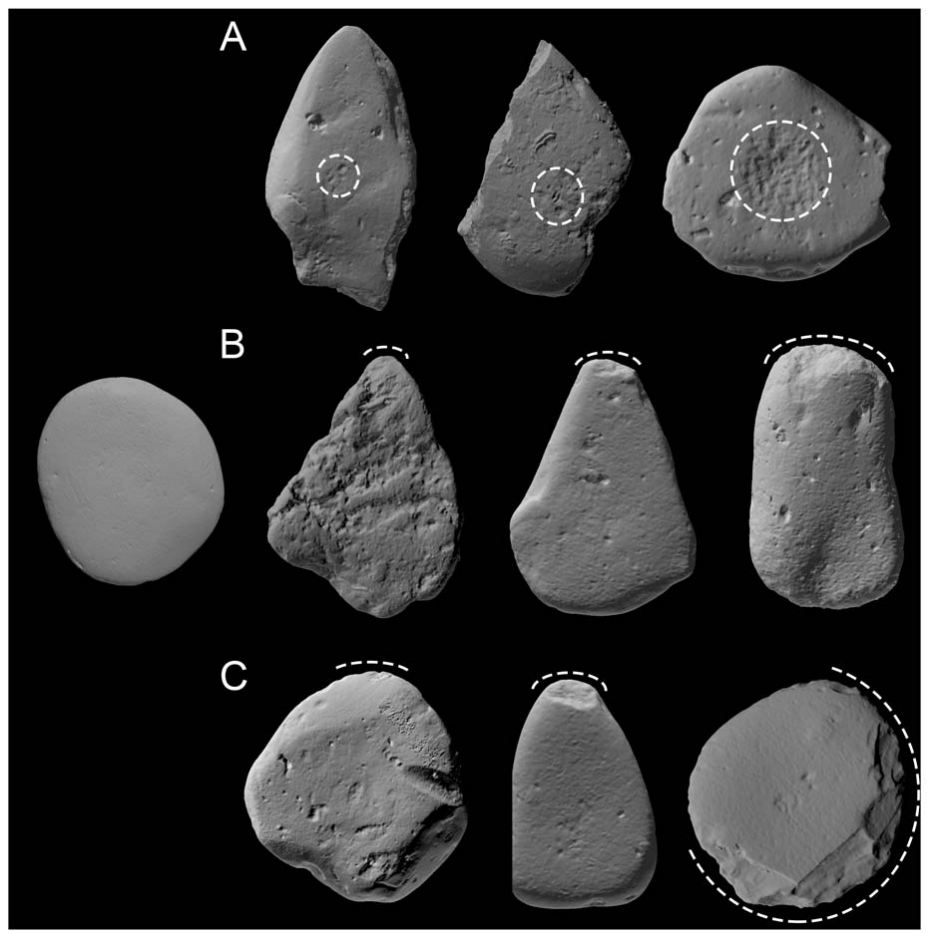
Examples of the pitting, crushing and fracture wear types. In each row, the wear intensity is shown from 1 (left) to 3 (right) on the 3D scan, with the worn portion of the tool highlighted by the dashed lines. The solitary stone at the left of the figure provides an undamaged comparison. A: Pitting wear on tools Skp01, Del01 and Nik02. B: Crushing wear on tools Gol01, Kiy02 and Gol04. C: Fracture wear on tools RF001, Sid01 and Med01. The scans are not at the same scale, and are shown at a similar size to demonstrate the relative extent of the damage. Metric data for each tool are provided in Table 1.

Each zone was scored for the presence of four wear types. The four types were: (i) *crushing* - abrasion of the stone surface resulting from impact, including rounding and levelling of protruding parts of the tool; (ii) *pitting* - discrete indentations from individual strikes that may result in cumulative creation of wide depressions in the stone surface; (iii) *fracture* - removal of sections of the stone through breakage, such as chipping or flaking; and (iv) *striations* - narrow linear scars, usually clustered at the point of impact. Damage on a tool resulted either from the impact of the tool with a processed food item, or incidental contact with the underlying substrate, such as a rock or mangrove root. Both types of damage were considered to be use-wear in this study, as they both result from tool use. For each wear type the intensity of the wear was recorded on the following three-point scale per zone: (1) *trace* - superficial and isolated points of damage only, possibly incidental; (2) *moderate* – the stone surface was clearly damaged but this damage was spatially restricted with only minor evidence of repeated wear; and (3) *extensive* - cumulative damage that may cover a significant portion of the use-zone. Except for striations, which proved not to be useful in determining tool use-histories (see below), the types of wear and examples of the intensity of each are shown in Figure 4.

### 3D scanning of tools

We used a portable laser scanner (NextEngine 3D Scanner HD with ScanStudio HD Pro software) to create a digital record of each tool, typically at a resolution of 40,000 points per square inch. This digitisation assisted in those instances where unaided observations could not determine whether damage (such as superficial striations) had permanently altered the stone surface. The 3D scans also permitted clear illustration of damage patterns, and archiving of the tool form data for future analyses.

### Statistical analyses

To identify the wear types and locations that were significant predictors of the observed use of the tools as axe or pound hammers, we performed binary logistic regression analyses on our complete dataset, as well as separately for each of the four wear types. Mann-Whitney U Tests were used to compare dimensions (weight and length) of stones classified as pound and axe hammers during direct observation, and to compare the intensity of use wear damage on tools of different stone materials (basalt and sandstone) and observed use-actions. A chi-squared test was used to test for differences in the proportion of stone materials comprising our pound and axe hammer samples. Cohen’s kappa coefficient was used to assess concordance between directly observed and predicted use-actions. Non-parametric tests were used for consistency throughout the paper, as some sample sizes were small, and therefore our data were unlikely to conform to the assumptions of parametric analyses. All statistical tests were two-tailed, with alpha set at 0.05.

### Ethics statement

The National Research Council of Thailand and the Department of National Parks, Wildlife and Plant Conservation of Thailand provided SM, MG, MH and DB with permits to carry out research activities at Laem Son National Park (LSNP). The methods used to collect stone tools from macaques in Thailand were reviewed and approved by the NTU-Institutional Animal Care & Use Committee (IACUC) in Singapore (ARF SBS/NIE-A 0138 AZ).

## Results

### Tool weights and lengths

The sample of 60 tools ranged from 32 g to 202 g and from 38 mm to 91 mm in length for axe hammers, and 10 g to 2434 g and 51 mm to 235 mm in length for pound hammers (Table 1). The pound hammers were significantly larger than the axe hammers in both weight and length (Mann-Whitney U-test, n_1_ = 42 n_2_ = 18: weight U = 637.5, p<0.001; length U = 634, p<0.001), replicating previous findings [20].

**Table 1:** Macaque stone tool materials, dimensions and weights.

Artefact	Stone	L (mm)	W (mm)	T (mm)	Wt (g)	Artefact	Stone	L (mm)	W (mm)	T (mm)	Wt (g)
**Amb01**	BA	99.16	68.22	30.22	176	**Luc04**	BA	114.72	62.29	33.31	216
**Amb02**	BA	190	92.63	29.27	834	**Luc05**	BA	135.57	47.85	17.82	174
**Amb03**	BA	119.24	75.93	25.97	376	**Luc06**	BA	143.21	77.74	31.75	390
**Cer01**	BA	154.25	74.41	30.43	358	**Luc07**	SI	127.73	70.56	41.73	478
**Che01**	BA	85.35	46.69	19	84	**Luc08**	BA	75.04	50.46	31.53	118
**Chr01**	SA	56.63	43.18	37.15	134	**Med01**	BA	61.39	54.49	15.11	66
**Del01**	BA	107.23	55.78	40.49	376	**Mrl01**	BA	71.15	28.8	18.24	62
**Del02**	BA	59.22	38.88	17.04	46	**Nef/Hly01**	BA	76.04	65.31	33.25	220
**Dnt01**	BA	65.97	56.49	29.88	150	**Nef02**	BA	50.45	35.53	12	36
**Drg01**	BA	215	178	45.23	2434	**Nik02**	BA	127.46	119.6	27.64	580
**Drs01**	BA	110.11	78.1	28.74	364	**Ogr01**	SA	58.6	47.36	22.79	66
**Els01**	BA	170	96.09	55.04	542	**Orc01**	BA	114.55	78.48	15.08	18
**Ezr01**	BA	72.48	58.29	28.7	172	**Pat01**	BA	169	103.47	53.3	1646
**Gol01**	QZ	69.16	52.97	33.32	140	**Ram02**	BA	108.44	67.89	25.42	264
**Gol02**	SA	71.21	45.09	35.49	150	**RamUn**	BA	176	82.43	33.88	824
**Gol03**	SA	58.14	44.57	21.34	62	**RF001**	BA	156	143.5	29.65	1146
**Gol04**	BA	66.5	36.18	19.14	66	**RF002**	BA	213	108.2	36.7	1386
**Hly01**	SA	60.87	49.8	22.01	104	**Sat01**	BA	87.15	64.62	20.37	178
**Icb01**	BA	175	75.77	37.81	624	**Sat02**	BA	108.83	68.35	25.97	272
**Inc02**	SA	77.35	66.53	33.72	210	**Scr01**	SA	109.91	88.65	47.74	528
**Inc06**	BA	62.08	19.06	19.01	40	**Scr02**	BA	46.26	34.05	12.23	32
**Inc08**	SA	74.42	46.71	13.72	62	**Sid01**	BA	57.09	35.28	15.94	58
**Inc09**	SA	59.19	27.49	21.54	44	**Skp01**	BA	191	102.55	36.9	852
**Ivy01**	BA	113.63	94.24	44.17	692	**Sln01**	BA	235	220	38.58	2406
**Jad01**	BA	90.93	74.45	20.03	202	**Sln02**	BA	154.22	120.64	37.99	1320
**Kiy02**	BA	68.08	39.75	15.76	64	**Smt01**	SA	38.01	32.02	29.22	34
**Lrl01**	BA	221	135.31	39.86	1872	**Tik01**	QZ	51.82	32.45	30.52	58
**Luc01**	SA	51.17	27.7	17.42	10	**Zar01**	BA	104.3	87.06	54.52	820
**Luc02**	BA	50.91	27.38	17.66	20	**Zar02**	SA	56.27	47.15	31.06	142
**Luc03**	SA	56.68	47.72	29.91	80	**Zar03**	SA	73.98	48.32	30.56	96

*Artefact*: Each alphanumeric code is comprised of a three-character identifier of the monkey that used the tool, followed by a number that separates different tools used by the one animal. *Stone*: SA = sandstone, BA = basalt, QZ = quartz, SI = siltstone. L = maximum length, W = width at 50% of the tool length, T = thickness at 50% of the tool length, Wt = weight.

### Use-Action Index

The complete use-wear dataset for all the analysed tools is provided in Table S1. Binary logistic regression analyses revealed that crushing wear on the tool points and faces were significant predictors of observed axe or pound hammer classification (crushing on points, p = 0.001; crushing on faces, p = 0.009). A simplified model that included only these two variables was able to predict observed use-actions with an accuracy of 90% (54 of 60 use-actions correctly predicted; odds ratios for crushing on points and faces were 0.27 and 3.41 respectively; Nagelkerke’s pseudo-R^2^ = 0.73). However, as there is a significant difference in tool weights between axe and pound hammers, we hypothesised that adding this variable may serve to increase the accuracy of our predictions. A binary logistic regression using the three variables showed that crushing remained a significant predictor, while tool weight did not reach significance (crushing on points, p = 0.005, odds ratio = 0.34; crushing on faces, p = 0.018, odds ratio = 3.40; tool weight, p = 0.158, odds ratio = 1.01). Nevertheless, including the weight variable slightly increased the predictive accuracy of the model, resulting in a match of predicted to observed use-action of 91.6% (55 of 60 use-actions correctly predicted; Nagelkerke’s pseudo-R^2^ = 0.81; Table 2).

**Table 2:** Use-Action Index, predicted use-action and last observed use-action.

Artefact	UAI	Pre	Obs	Artefact	UAI	Pre	Obs	Artefact	UAI	Pre	Obs
**Amb01**	–6.13	P	P	**Inc06**	–3.31	P	P	**Ogr01**	–3.37	P	A
**Amb02**	–15.35	P	P	**Inc08**	0.04	A	A	**Orc01**	–3.00	P	P
**Amb03**	–7.86	P	P	**Inc09**	2.73	A	A	**Pat01**	–26.71	P	P
**Cer01**	–9.90	P	P	**Ivy01**	–13.36	P	P	**Ram02**	–9.81	P	P
**Che01**	0.95	A	A	**Jad01**	1.44	A	A	**RamUn**	–10.33	P	P
**Chr01**	0.25	A	P	**Kiy02**	3.37	A	A	**RF001**	–16.05	P	P
**Del01**	–5.27	P	P	**Lrl01**	–31.10	P	P	**RF002**	–17.42	P	P
**Del02**	2.56	A	A	**Luc01**	–2.59	P	P	**Sat01**	–4.02	P	P
**Dnt01**	1.87	A	A	**Luc02**	0.63	A	P	**Sat02**	–5.04	P	P
**Drg01**	–35.31	P	P	**Luc03**	–1.13	P	P	**Scr01**	–8.62	P	P
**Drs01**	–5.26	P	P	**Luc04**	–6.69	P	P	**Scr02**	2.90	A	A
**Els01**	–8.82	P	P	**Luc05**	–4.89	P	P	**Sid01**	1.02	A	P
**Ezr01**	–6.08	P	P	**Luc06**	–9.13	P	P	**Skp01**	–15.60	P	P
**Gol01**	1.39	A	A	**Luc07**	–10.36	P	P	**Sln01**	–37.35	P	P
**Gol02**	3.09	A	A	**Luc08**	–1.96	P	P	**Sln02**	–22.15	P	P
**Gol03**	–1.18	P	P	**Med01**	5.49	A	A	**Smt01**	1.80	A	A
**Gol04**	5.49	A	A	**Mrl01**	5.54	A	A	**Tik01**	2.54	A	A
**Hly01**	–0.55	P	A	**Nef/Hly01**	–3.09	P	P	**Zar01**	–13.93	P	P
**Icb01**	–8.75	P	P	**Nef02**	2.55	A	A	**Zar02**	–0.78	P	P
**Inc02**	–1.73	P	P	**Nik02**	–9.50	P	P	**Zar03**	–1.35	P	P

*UAI*: Use-Action Index, see text for details. *Pre*: use-action predicted by UAI (A = axe hammer, P = pound hammer). *Obs*: observed use-action prior to tool collection.

We propose that the resulting equation, which we have named the Use-Action Index (UAI), can therefore assist in distinguishing between axe and pound hammers at PNY. It is expressed as: UAI  =  1.21 + (1.07×Cp) – (1.22×Cf) – (0.014×Wt). In this index, for each tool Cp is the total combined intensity value for crushing on the tool point, Cf is the combined crushing value for the tool face, and Wt is the tool weight in grams. A positive UAI value derives from greater intensity of crushing on the tool point and a lower relative weight, and results in classification of a tool as an axe hammer. Conversely, tools with a negative UAI derive this value from greater intensity of crushing on the tool face and higher relative weight, and these were classified as pound hammers. A Cohen’s kappa value of 0.80 demonstrates that agreement between the UAI and directly observed data is unlikely to have resulted from chance.

**Figure 5:**
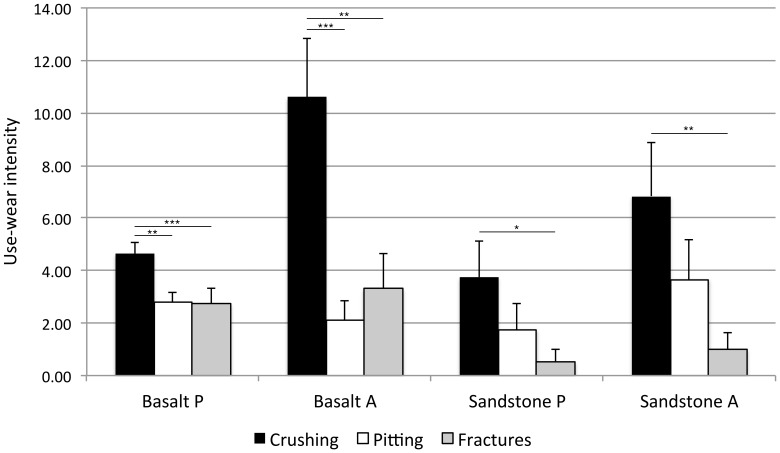
Mean use-wear intensity per tool. Bars denote standard errors. P  =  pound hammer, A  =  axe hammer. * denotes significance at p<0.05, ** p<0.01 and *** p<0.001.

Of the four stone materials included in this study, our sample contained predominantly basalt (n = 43) and sandstone (n = 14), with siltstone (n = 1) and quartz (n = 2) also present. Basalt tools in this sample were used significantly more than expected for pounding activities, when compared to basalt axe hammers and sandstone tools (chi-squared test, p = 0.018). We also compared the average intensity of crushing, pitting and fracture wear per tool for the basalt and sandstone artefacts, excluding the other materials because of insufficient sample size. The basalt and sandstone tools were further separated into those that were directly observed being used as axe or pound hammers, to allow us to identify any differences in the intensity of use-damage across these use-actions. Figure 5 shows the results of this analysis, demonstrating that there is significantly greater crushing wear intensity than pitting or fracture wear on basalt pound and axe hammers (basalt pound hammers, n = 33: crushing vs pitting, p = 0.002, U = 781.5; crushing vs fractures, p<0.001, U = 798.5; basalt axe hammers, n = 10: crushing vs pitting, p<0.001, U = 92.5; crushing vs fractures, p = 0.003, U = 87.5). There is also significantly more crushing wear than fractures on sandstone pound and axe hammers (sandstone pound hammers, n = 8: crushing vs fractures, p = 0.038, U = 52.0; sandstone axe hammers, n = 6: crushing vs fractures, p = 0.009, U = 34.0). All other relative wear intensities within these tool classes are not significantly different. These results suggest that on basalt and sandstone artefacts crushing wear either (i) develops more rapidly than other wear types during their use by the wild macaques, or (ii) is better preserved than other wear types at PNY. In either instance these results present further justification for including crushing use-wear in the UAI.

## Discussion

Based on a combination of use-wear type, intensity and location on each tool, as well as tool weight, the Use-Action Index was able to differentiate axe and pound hammers used by wild Burmese long-tailed macaques with a success rate of 92%. These results confirm that *M. f. aurea* tool use leaves identifiable and interpretable wear traces on stone artefacts, which is promising for the future archaeological recovery and analysis of tools used by these monkeys, and for comparison between macaque tools, those of other wild stone-tool-using primates, and hominin pounding tools.

**Figure 6:**
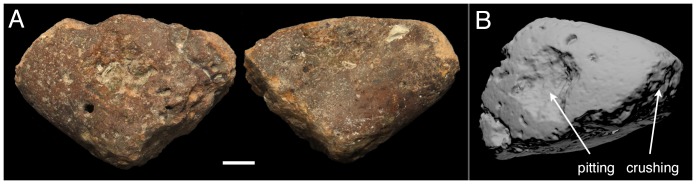
A versatile tool. Gol02 displays extensive pitting wear on the faces and crushing on both the point and base. A: photograph with 1 cm scale; B: 3D scan. Photos by MG and MH.

Five tools had a predicted use-action that did not match the directly observed use of that tool as an axe or pound hammer (Chr01, Hly01, Luc02, Ogr01 and Sid01). Three of these (Chr01, Luc02 and Sid01) were classified as axe hammers by the UAI, but were observed being used as pound hammers, and for two tools (Hly01 and Ogr01) the reverse situation applied. The UAI values for these mis-classified tools ranged from –3.37 to 1.02, close to the zero value that divides axe from pound hammers (UAI values for the sample as a whole range from –37.35 to 5.54). Except for Ogr01, these tools had either equal values for crushing wear intensity on the tool point and face, or a single point different between the two areas. The UAI may not therefore have sufficient discriminatory power in such borderline cases, although we note that it was successful in predicting the use-action of tools that exhibit different types of wear on the face and point, such as Gol02 (Figure 6). As monkeys at PNY have been observed to occasionally use the same tool for axe and pound hammering (M. Gumert, unpublished data), this may also act to obscure behavioural reconstructions. The final mis-classified tool, Ogr01, showed evidence of crushing on the face but not the point, despite being observed in use as an axe hammer. In such cases, where diagnostic use-damage is not created at the point of contact, a tool’s behavioural history is unlikely to be recoverable through any form of use-wear analysis. Microscopic residue analysis of plant or animal remains adhering to the tool [3], [33], [34] may be of benefit in such instances.

In future, we intend to further refine the use-wear intensity scale developed in this study to permit its use in interpreting archaeologically recovered tools that have been used by Burmese long-tailed macaques, provided such tools can be reliably distinguished from those of other animals and humans, and from environmentally-damaged stones. We expect that such differentiation may be possible based on species distributions, tool sizes, use-wear patterns, and the landscape distribution of tools and food debris, but studies to test this expectation have yet to be conducted. While we predict that low intensity (trace) wear on the macaque tools may not be recognisable after weathering, trampling or extended burial, both moderate and extensive wear more lastingly modify the stone surface. The rate of loss of use-wear on tools exposed to factors such as the tidal, rainfall and wave conditions seen at PNY will need to be assessed in future analyses to determine the durability of damage patterns, which are dependent also on the integrity of the stone tool surface. However, the prevalence of crushing wear, the hardness of the encased coastal resources exploited by the macaques on PNY, the use of durable tool materials such as basalt, and the repeated pounding actions employed, are all favourable indicators for the long-term survival and integrity of Burmese long-tailed macaque artefacts. With increased sample sizes in the future, we may also be able to develop material-specific UAIs that improve the accuracy of our behavioural reconstructions. At the same time, the processes and rate of use-wear formation also require study, including factors influencing damage patterns such as (i) the tool user’s age and sex, (ii) the hardness and composition of stones used as tools, and (ii) the hardness and shape of processed foods.

Continued study of use-wear patterns generated by the PNY macaques will increase the likelihood of correctly identifying macaque tool use sites where this behaviour was previously practiced but is not currently present (either through local extinction of macaque groups or a temporal break in their tool use traditions). The use of stone tools by Burmese long-tailed macaques was first reported in the coastal regions of Burma, north of Laem Son National Park, in the 1880s [24], and along with anecdotal reports of present day tool use in east and west coastal Thailand [26] this suggests that this behaviour previously had a more geographically extensive southeast Asian distribution. We hypothesise, therefore, that there are a number of as-yet unidentified macaque tool use sites along the Thailand and Burmese coasts, with site locations and the spread of tool use by past macaque populations influenced by the dramatic sea level fluctuations of recent glacial periods [35], [36]. A primary means of identifying such sites will be via the recovery, through archaeological survey and excavation, of stones of a size and material suitable for macaque stone pounding. The interpretation of the behavioural history of such stones will rely heavily on use-wear evidence.

## Conclusion

We have demonstrated that use-wear analysis can successfully classify stone artefacts used with two different actions by wild Burmese long-tailed macaques. This finding demonstrates that use-wear patterns can reliably reveal the previous history of macaque stone tool behaviour. These data will inform ongoing research into the hand grips, striking precision, and tool orientations used by wild macaques both in the present day and in the past. We anticipate that the methods employed here can be fruitfully applied to other macaque sites in Thailand, and potentially to cross-taxa comparisons with other primate and hominin species. Further development and refinement of quantitative analytical techniques, such as the UAI, is necessary to allow primate archaeology to extend our knowledge of non-hominin technological and behavioural evolution.

## Supporting Information

Table S1 **Use-wear intensity data.**
(DOCX)
