# Supplementary material for: Use-Wear Patterns on Wild Macaque Stone Tools Reveal Their Behavioural History
Source: PLoS One. 2013 Aug 16;8(8):e72872. doi: 10.1371/journal.pone.0072872 (PMC3745380; doi:10.1371/journal.pone.0072872)
Supplement: Table S1 — Use-wear intensity data. (DOCX) [file pone.0072872.s001.docx]

**Table S1. Use-wear intensity data**

|  | **Crushing** | | | | **Pitting** | | | | **Fractures** | | | | **Striations** | | | |
| --- | --- | --- | --- | --- | --- | --- | --- | --- | --- | --- | --- | --- | --- | --- | --- | --- |
| **Tool** | **Point** | **Edge** | **Face** | **Base** | **Point** | **Edge** | **Face** | **Base** | **Point** | **Edge** | **Face** | **Base** | **Point** | **Edge** | **Face** | **Base** |
| Amb01 | 0 | 2 | 4 | 1 | 0 | 0 | 3 | 1 | 0 | 0 | 0 | 0 | 0 | 1 | 0 | 0 |
| Amb02 | 0 | 0 | 4 | 0 | 0 | 0 | 4 | 0 | 0 | 1 | 0 | 0 | 0 | 0 | 0 | 0 |
| Amb03 | 1 | 1 | 4 | 0 | 0 | 0 | 4 | 0 | 0 | 0 | 0 | 0 | 0 | 0 | 1 | 0 |
| Cer01 | 0 | 0 | 5 | 0 | 0 | 0 | 3 | 0 | 0 | 3 | 0 | 3 | 0 | 0 | 0 | 0 |
| Che01 | 2 | 0 | 1 | 0 | 0 | 0 | 1 | 0 | 4 | 3 | 0 | 0 | 0 | 0 | 0 | 0 |
| Chr01 | 2 | 4 | 1 | 2 | 0 | 1 | 0 | 2 | 0 | 0 | 0 | 0 | 0 | 0 | 0 | 0 |
| Del01 | 0 | 0 | 1 | 0 | 0 | 0 | 2 | 0 | 0 | 0 | 0 | 0 | 0 | 0 | 0 | 0 |
| Del02 | 3 | 0 | 1 | 0 | 0 | 0 | 0 | 0 | 1 | 0 | 0 | 0 | 0 | 0 | 0 | 1 |
| Dnt01 | 6 | 9 | 3 | 4 | 4 | 1 | 0 | 2 | 1 | 0 | 0 | 0 | 0 | 0 | 3 | 0 |
| Drg01 | 0 | 2 | 2 | 1 | 0 | 0 | 0 | 0 | 1 | 0 | 0 | 0 | 0 | 0 | 0 | 0 |
| Drs01 | 1 | 0 | 2 | 0 | 0 | 0 | 4 | 0 | 0 | 0 | 0 | 0 | 0 | 0 | 0 | 0 |
| Els01 | 0 | 0 | 2 | 0 | 0 | 0 | 0 | 0 | 1 | 0 | 0 | 0 | 0 | 0 | 0 | 0 |
| Ezr01 | 0 | 0 | 4 | 0 | 0 | 0 | 1 | 0 | 0 | 0 | 0 | 0 | 0 | 0 | 4 | 2 |
| Gol01 | 2 | 1 | 0 | 2 | 0 | 0 | 0 | 0 | 2 | 0 | 0 | 0 | 0 | 0 | 0 | 0 |
| Gol02 | 6 | 4 | 2 | 4 | 2 | 2 | 5 | 1 | 0 | 0 | 0 | 0 | 0 | 0 | 0 | 0 |
| Gol03 | 2 | 2 | 3 | 3 | 0 | 2 | 6 | 0 | 1 | 1 | 0 | 2 | 0 | 0 | 2 | 0 |
| Gol04 | 6 | 2 | 1 | 5 | 1 | 2 | 0 | 0 | 2 | 0 | 0 | 1 | 0 | 0 | 0 | 0 |
| Hly01 | 2 | 4 | 2 | 1 | 0 | 0 | 5 | 0 | 0 | 0 | 0 | 0 | 0 | 0 | 0 | 0 |
| Icb01 | 0 | 1 | 1 | 0 | 0 | 0 | 0 | 0 | 5 | 4 | 0 | 4 | 0 | 0 | 0 | 0 |
| Inc02 | 0 | 0 | 0 | 0 | 0 | 0 | 0 | 0 | 0 | 0 | 0 | 0 | 0 | 0 | 0 | 0 |
| Inc06 | 2 | 0 | 5 | 0 | 0 | 0 | 4 | 0 | 0 | 0 | 0 | 0 | 0 | 1 | 0 | 0 |
| Inc08 | 2 | 0 | 2 | 2 | 0 | 0 | 3 | 0 | 0 | 3 | 0 | 0 | 0 | 0 | 0 | 0 |
| Inc09 | 2 | 0 | 0 | 2 | 0 | 0 | 0 | 0 | 0 | 0 | 0 | 0 | 0 | 0 | 0 | 0 |
| Ivy01 | 0 | 1 | 4 | 0 | 0 | 0 | 4 | 0 | 0 | 1 | 0 | 0 | 0 | 0 | 1 | 0 |
| Jad01 | 4 | 2 | 1 | 0 | 0 | 0 | 2 | 0 | 2 | 0 | 0 | 0 | 0 | 0 | 1 | 0 |
| Kiy02 | 4 | 2 | 1 | 0 | 0 | 0 | 0 | 0 | 1 | 1 | 0 | 1 | 0 | 0 | 1 | 0 |
| Lrl01 | 0 | 1 | 5 | 1 | 0 | 0 | 5 | 0 | 3 | 2 | 0 | 0 | 0 | 0 | 1 | 1 |
| Luc01 | 0 | 0 | 3 | 0 | 0 | 0 | 0 | 0 | 0 | 0 | 0 | 0 | 0 | 0 | 1 | 0 |
| Luc02 | 2 | 4 | 2 | 0 | 0 | 2 | 0 | 0 | 4 | 0 | 0 | 0 | 0 | 0 | 1 | 0 |
| Luc03 | 0 | 4 | 1 | 0 | 0 | 0 | 0 | 0 | 0 | 0 | 0 | 0 | 0 | 0 | 0 | 0 |
| Luc04 | 0 | 0 | 4 | 0 | 0 | 0 | 0 | 0 | 2 | 6 | 0 | 0 | 0 | 0 | 0 | 0 |
| Luc05 | 0 | 0 | 3 | 0 | 0 | 0 | 0 | 0 | 0 | 1 | 0 | 0 | 0 | 0 | 3 | 0 |
| Luc06 | 0 | 0 | 4 | 0 | 0 | 0 | 3 | 0 | 0 | 0 | 0 | 0 | 0 | 0 | 2 | 0 |
| Luc07 | 0 | 1 | 4 | 0 | 0 | 0 | 5 | 0 | 0 | 1 | 0 | 0 | 0 | 0 | 2 | 0 |
| Luc08 | 2 | 3 | 3 | 2 | 0 | 0 | 4 | 0 | 0 | 0 | 0 | 2 | 0 | 1 | 2 | 0 |
| Med01 | 6 | 10 | 1 | 5 | 0 | 0 | 1 | 0 | 6 | 6 | 0 | 2 | 0 | 0 | 2 | 0 |
| Mrl01 | 6 | 0 | 1 | 4 | 1 | 0 | 1 | 0 | 0 | 0 | 0 | 0 | 0 | 0 | 0 | 0 |
| Nef/Hly01 | 0 | 0 | 1 | 0 | 0 | 0 | 2 | 0 | 0 | 0 | 0 | 0 | 0 | 0 | 0 | 0 |
| Nef02 | 4 | 0 | 2 | 6 | 2 | 0 | 0 | 3 | 0 | 0 | 0 | 0 | 0 | 0 | 1 | 0 |
| Nik02 | 1 | 0 | 3 | 0 | 0 | 0 | 4 | 0 | 0 | 10 | 0 | 0 | 0 | 0 | 0 | 0 |
| Ogr01 | 0 | 0 | 3 | 0 | 0 | 0 | 4 | 0 | 0 | 0 | 0 | 3 | 0 | 0 | 0 | 0 |
| Orc01 | 2 | 0 | 5 | 0 | 0 | 0 | 7 | 0 | 0 | 0 | 0 | 0 | 0 | 0 | 2 | 0 |
| Pat01 | 0 | 3 | 4 | 0 | 0 | 0 | 0 | 0 | 1 | 3 | 0 | 0 | 0 | 0 | 2 | 0 |
| Ram02 | 0 | 0 | 6 | 0 | 0 | 0 | 4 | 0 | 4 | 7 | 0 | 0 | 0 | 0 | 0 | 0 |
| RamUn | 0 | 0 | 0 | 0 | 0 | 2 | 5 | 1 | 0 | 0 | 0 | 0 | 0 | 0 | 0 | 0 |
| RF001 | 0 | 0 | 1 | 0 | 0 | 0 | 2 | 0 | 1 | 2 | 0 | 2 | 0 | 0 | 0 | 0 |
| RF002 | 3 | 0 | 2 | 0 | 0 | 0 | 4 | 0 | 0 | 1 | 0 | 0 | 0 | 0 | 1 | 0 |
| Sat01 | 2 | 0 | 4 | 0 | 0 | 0 | 0 | 0 | 0 | 2 | 0 | 0 | 0 | 0 | 1 | 0 |
| Sat02 | 0 | 0 | 2 | 0 | 0 | 0 | 2 | 0 | 1 | 0 | 0 | 0 | 0 | 0 | 0 | 0 |
| Scr01 | 0 | 0 | 2 | 0 | 0 | 0 | 2 | 0 | 0 | 0 | 0 | 0 | 0 | 0 | 0 | 0 |
| Scr02 | 2 | 0 | 0 | 2 | 0 | 0 | 0 | 0 | 2 | 0 | 0 | 0 | 0 | 0 | 0 | 0 |
| Sid01 | 4 | 1 | 3 | 0 | 0 | 0 | 2 | 0 | 2 | 0 | 0 | 0 | 0 | 0 | 0 | 0 |
| Skp01 | 0 | 3 | 4 | 0 | 0 | 0 | 4 | 0 | 0 | 4 | 1 | 0 | 0 | 1 | 1 | 0 |
| Sln01 | 0 | 0 | 4 | 0 | 0 | 1 | 4 | 0 | 1 | 2 | 0 | 0 | 0 | 0 | 0 | 0 |
| Sln02 | 0 | 0 | 4 | 0 | 0 | 0 | 0 | 0 | 2 | 1 | 0 | 0 | 0 | 0 | 2 | 0 |
| Smt01 | 1 | 2 | 0 | 0 | 0 | 0 | 0 | 0 | 0 | 0 | 0 | 0 | 0 | 0 | 0 | 0 |
| Tik01 | 2 | 0 | 0 | 0 | 0 | 0 | 0 | 0 | 2 | 0 | 0 | 0 | 0 | 0 | 0 | 0 |
| Zar01 | 0 | 0 | 3 | 1 | 0 | 0 | 3 | 1 | 0 | 0 | 0 | 0 | 0 | 0 | 0 | 0 |
| Zar02 | 0 | 0 | 0 | 0 | 0 | 0 | 0 | 0 | 0 | 0 | 0 | 0 | 0 | 0 | 0 | 0 |
| Zar03 | 0 | 0 | 1 | 0 | 0 | 0 | 1 | 0 | 0 | 0 | 0 | 0 | 0 | 0 | 0 | 0 |
